# Ab-initio study of pressure influenced elastic, mechanical and optoelectronic properties of Cd_0.25_Zn_0.75_Se alloy for space photovoltaics

**DOI:** 10.1038/s41598-022-17218-8

**Published:** 2022-07-28

**Authors:** Muhammad Aamir Iqbal, Maria Malik, Wajeehah Shahid, Shaheen Irfan, Arnold C. Alguno, Kareem Morsy, Rey Y. Capangpangan, Phuong V. Pham, Jeong Ryeol Choi

**Affiliations:** 1 Centre of Excellence in Solid State Physics, University of the Punjab, Lahore, 54590 Pakistan; 2 School of Materials Science and Engineering, Zhejiang University, Hangzhou, 310027 China; 3 Department of Physics, The University of Lahore, Lahore, 54000 Punjab Pakistan; 4 Department of Physics, Premier Research Institute of Science and Mathematics (PRISM), Mindanao State University - Iligan Institute of Technology, Tibanga Highway, 9200 Iligan City, Philippines; 5 Biology Department, College of Science, King Khalid University, Abha, 61321 Saudi Arabia; 6 Zoology Department, Faculty of Science, Cairo University, Cairo, 11351 Egypt; 7 Department of Physical Sciences and Mathematics, College of Science and Environment, Mindanao State University at Naawan, 9023 Naawan, Misamis Oriental Philippines; 8 Hangzhou Global Scientific and Technological Innovation Center, School of Micro-Nano Electronics, Zhejiang University, Hangzhou, 310027 China; 9 Department of Nanoengineering, Kyonggi University, Suwon, 16227 South Korea

**Keywords:** Materials science, Electronic devices

## Abstract

The optoelectronic properties of the ternary Cd_0.25_Zn_0.75_Se alloy are reported under the influence of a high pressure ranging from 0 to 25 GPa, within a modified Becke–Jhonson potential using density functional theory. This alloy has a cubic symmetry, is mechanically stable, and its bulk modulus rises with pressure. It is observed to be a direct bandgap material with a bandgap energy that increases from 2.37 to 3.11 eV with rise in pressure. Pressure changes the optical and electronic properties, causing the absorption coefficient to rise and absorb visible green-to-violet light. The static dielectric constant, along with the static index of refraction, both increase under the influence of pressure. Optical constants, including dielectric constant, optical conductivity, refractive index, extinction coefficient, and reflection, are also investigated and discussed. This DFT forecast explores important research directions for the usage of the CdZnSe semiconductor alloys in the manufacturing of space photovoltaic and optoelectronic devices operating at different pressures.

## Introduction

The use of updated technology continuously leads to further technological innovations where the rapid growth of ternary alloys, as well as the inclusion of new application fields and technical advancement, poses several scientific and technological challenges. By adjusting the composition and eliciting pressure effects, the properties of semiconductors in groups II–VI may be tailored for specific uses in well-known marketed optoelectronic devices that can function over whole spectrum ranges^1^. The direct bandgap of these alloys plays a key role in numerous intriguing device applications, including the optoelectronic and photovoltaic industries, owing to their tunable bandgap under the influence of composition and pressure^2^. Variable wavelength photodetectors, light emitting diodes, light sensors, solar cells, space photovoltaics, and organic materials-based like devices are all possible applications for these ternary alloys^2–8^.

CdZnSe ternary alloys are of great interest and found to be appealing for use in the production of photoluminescent, photoconductive, and luminescent devices due to their higher stability and wide bandgap^9–12^. Thin films of CdZnSe semiconductors have been synthesized to study structural, dielectric, and magnetic properties by molecular beam epitaxy (MBE)^13^, electro-deposition^14^, vacuum evaporation^15^, and chemical bath deposition (CBD) techniques^12^. These studies have been performed for structural properties^16,17^, dielectric properties^18^, and magnetic properties^19^. The synthesis of CdZnSe quantum dots has also been reported by Loghina et al. in which they have measured a direct bandgap of 2.27 eV^20^. Theoretically, electronic and optical features have been investigated using a plane-wave pseudopotential approach without pressure treatment within the CASTEP code^21^, while the thermodynamic characteristics of ternary Cd_0.25_Zn_0.75_Se semiconductors have been explored within a theoretical model for the temperature range of 0–1000 K and a pressure of 0–10 GPa, respectively, using Quantum Expresso software^22^. Certain physical properties at ambient pressure, including electronic and structural characteristics, have also been analyzed using the first-principles method^23^.

According to our understanding, there is a significant shortcoming in the behavior of the selected ternary Cd_0.25_Zn_0.75_Se alloy, and the lack of adequate information motivated us to explore its optoelectronic properties under the influence of high pressure. This study focuses on providing theoretical information on optoelectronic characteristics and analysis to understand the underlying physical phenomena that occur under the influence of high pressure. Structural property relationships and stability under high pressure are investigated for the first time and also discussed. In the present study, DFT method within mBJ potential was employed together to explore the elastic, electronic, mechanical, and optical properties of the ternary Cd_0.25_Zn_0.75_Se semiconductor at distinct hydrostatic pressures. It is an initial step to explore the optoelectronic characteristics of this material under the influence of high pressure.

## Theoretical method

The Kohn–Sham equations are solved with the help of first-principles calculations using density functional theory (DFT)^24^ as employed in the Wien2k software^25^. To employ this DFT approach, the core and valence states were set at an energy separation of − 6.0 Ry, while the spherical harmonic wave potentials for the core were extended to the value of L_max_ = 10. The convergence criteria were established to be 1 mRy/Bohr, 0.00001 e, and 0.00001 Ry for force, charge, and energy, respectively, where the R_MT_*K_max_ value was taken as 7.0. The radius of muffin-tin spheres (RMTs) for Cd, Zn, and Se atoms were set at 2.29, 2.29, and 2.18 Bohr, while a k-point mesh sampling was generated as 12 × 12 × 12 order in the irreducible Brillouin zone. The modified Becke–Jhonson method (mBJ), proposed by Tran and Blaha^26^, was used to investigate the optoelectronic properties as a function of pressure.

The effect of pressure on structural parameters such as lattice constant was approximated with the help of^27^; a(P) = a_0_[${1+P\frac{{B}^{{\prime}}}{{B}_{0}}]}^{\frac{-1}{{3B}^{{\prime}}}}$, in which a_0_ represents the lattice constant at ambient pressure, P is the hydrostatic pressure, B_0_ is the bulk modulus, and B^′^ represents the bulk modulus of pressure derivative, respectively. Numerous researches have already established the accomplishment of this relationship in assessing the varying pressure influence on the structural parameters and, as a consequence, on the other characteristics. Their respective outcomes are identical to the experimental results, giving us confidence in the consistency of employing this relationship to examine the influence of pressure on the optoelectronic characteristics of the Cd_0.25_Zn_0.75_Se alloy.

## Results and discussion

### Mechanical stability and elastic properties

**Figure 1:**
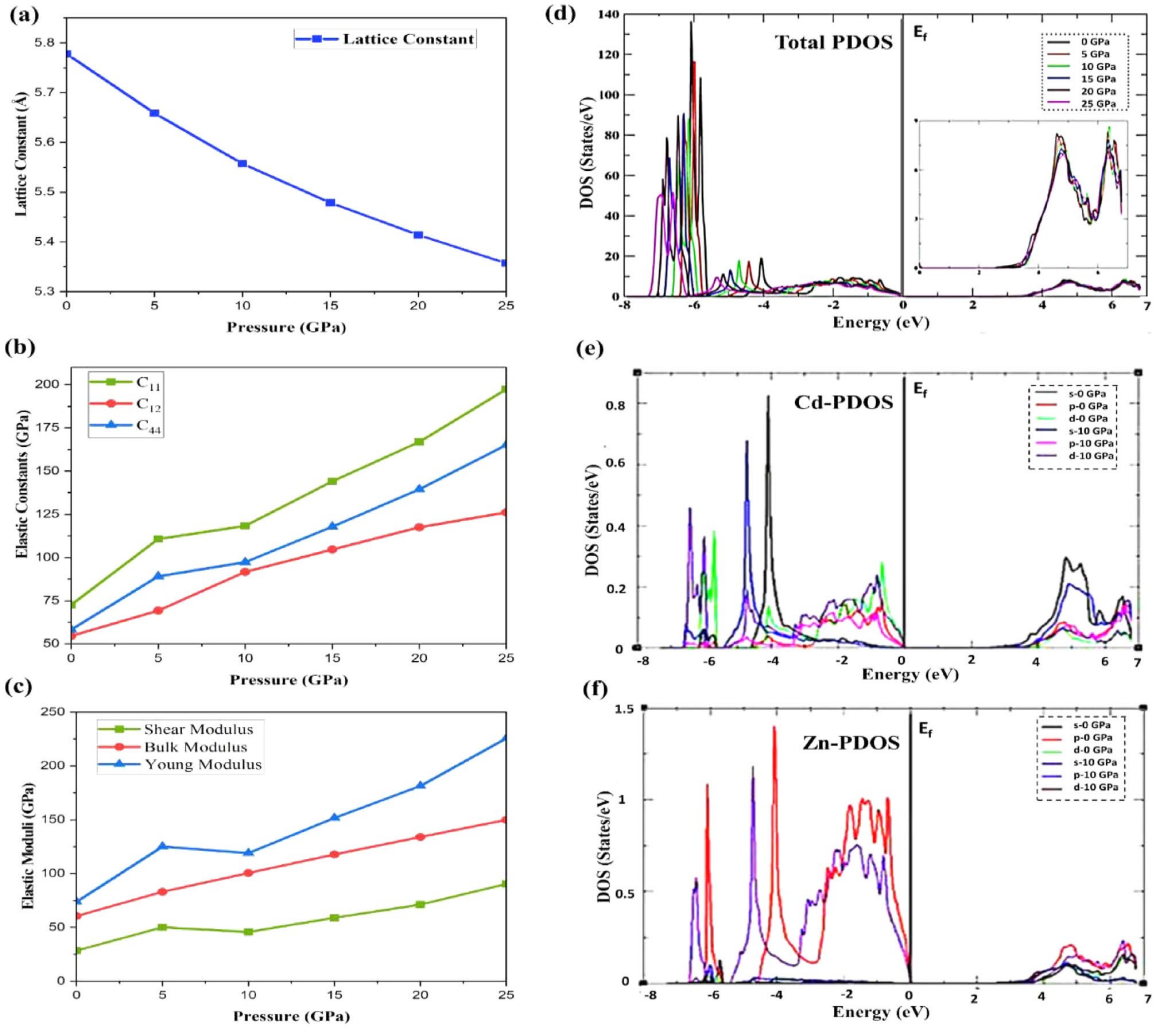
Elastic constants and density of states analysis under the influence of pressure, (**a**) lattice constant variation, (**b**) C_11_, C_12_, C_14_ variation, (**c**) shear moduls, bulk modulus, young modulus variation, (**d**) TDOS of Cd_0.25_Zn_0.75_Se alloy, (**e**) PDOS of Cd-atom, and (**f**) PDOS of Zn-atom.

At ambient pressure, the Cd_0.25_Zn_0.75_Se alloy exhibits a cubic zinc-blende structure. We calculated elastic constants at different pressures to investigate mechanical stability under the influence of high pressure, and it was found that the elastic constants increased with an increase in pressure and cubic stability conditions were satisfied. In cubic crystals, there exist only three independent elastic constants: C_11_, C_12_ and C_44_. The Born stability criteria are tested at ambient pressure for mechanical stability using C_11_–C_12_ > 0, C_44_ > 0, and C_11_ + 2C_12_ > 0, while at high pressure, the additional standards for the (mechanical) stability of structures are C_11_ + C_12_ + P > 0, C_11_–C_12_–2P > 0, and C_44_–P > 0^28^. The elastic constants under ambient and high pressure satisfy the above stability standards, and hence this ternary alloy is mechanically stable within the pressure range of 0–25 GPa. The lattice constant, elastic coefficients, and density of states variation under the high pressure’s influence are shown in Fig. 1a–c. A summary of elastic constants and stability parameters is presented in Table 1. According to the elastic moduli data, it is obvious that this alloy satisfies all stability criteria, is stable in cubic symmetry under high pressure, and can be potentially applied in device fabrication.

**Table 1:** Elastic moduli and lattice parameters along with stability criteria computed under the influence of pressure from 0 to 25 GPa.

Pressure (GPa)	Elastic constants			Lattice constant	Elastic moduli (GPa)	
	C_11_ (GPa)	C_12_ (GPa)	C_44_ (GPa)	a = b = c (Å)	Bulk modulus (GPa)	C_11_–C_12_–2P > 0 (GPa)
0	72.601	54.551	58.227	5.578	60.567	18.050
5	110.706	69.263	89.075	5.647	83.077	31.443
10	118.275	91.654	97.319	5.551	100.527	6.621
15	144.071	104.608	117.836	5.474	117.762	9.463
20	166.893	117.486	139.443	5.411	133.955	9.407
25	197.400	126.129	165.152	5.357	149.886	21.271

The mechanical properties of a material, including bulk modulus (B), shear modulus (G), Pugh ratio, anisotropy factor (A), Young’s modulus (E), Cauchy’s pressure (CP), and Poisson ratio (σ)^29,30^, are used to study the mechanical stability of investigated materials^31^. Bulk modulus (B) may be presented as:

1 $$B=\frac{{B}_{v}+{B}_{R}}{2}$$

wherein,

2 $${B}_{v}={B}_{R}=\frac{{C}_{11}+2{C}_{12}}{3}$$

and B_v_ represents Voigt bulk modulus, while B_R_ represents Reuss bulk modulus. However, the stiffness of material can also be determined by the Shear modulus (G) as;

3 $$G= \frac{{G}_{v}+{G}_{R}}{2}$$

wherein,

4 $${G}_{V}= \frac{1}{5} (3{C}_{44}+ {C}_{11}- {C}_{12})$$

and,

5 $${G}_{R}=\frac{5\left({C}_{11}- {C}_{12}\right){C}_{44}}{3\left({C}_{11}- {C}_{12}\right){+ 4C}_{44}}$$

where G_v_ represents Voigt shear modulus while G_R_ represents Reuss shear modulus^32^. Furthermore, Poisson ratio (σ), and young modulus (E) may be explained in terms of both bulk modulus and shear modulus as follows;

6 $$E= \frac{9GB}{3G+B}$$

7 $$\sigma = \frac{3B-G}{2 (3G+B)}$$

Cauchy’s pressure (CP) and anisotropic factor (A), can be mathematically illustrated as;

8 $$CP={C}_{12}- {C}_{44}$$

9 $$A= \frac{2{C}_{44}}{{C}_{11}- {C}_{12}}$$

Moreover, the Pugh’s ratio is described as the ratio of bulk modulus to shear modulus (B/G), to determine a material’s ductility or brittleness. A material’s value larger than 1.75 G/B ratio is considered a ductile material, while less than 1.75 G/B value is indicated as a brittle material^33,34^. The critical value of Pugh’s ratio is estimated to be greater than 1.75 at a pressure range of 10–20 GPa for CdZnSe, depicting it as a ductile material in this range while brittle at low pressure and above the 20 GPa pressure range. Cauchy’s pressure (CP) and Poisson ratio (σ) act in the same way to determine a material’s mechanical characteristics. When the CP of a material is positive, then the material is considered to have a ductile nature, whereas if the CP values are negative, then the material acts as brittle^35^. In the present study of CdZnSe, CP values are negative, which shows that the material is brittle under varying pressures. Furthermore, a material is said to be isotropic if its anisotropic factor (A) is one, otherwise it is anisotropic material. According to the values calculated in Table 2, CdZnSe is an anisotropic material, having an anisotropic factor greater than 1 with varying pressure from 0 to 25 GPa. Table 2 summarizes the mechanical properties, including bulk modulus, shear modulus, Pugh ratio, anisotropy factor, young’s modulus, Cauchy’s pressure, and Poisson ratio.

**Table 2:** Mechanical parameters of the CdZnSe alloy at various pressures ranging from 0 to 25 GPa.

Pressure (GPa)	Bulk modulus (B)	Shear modulus (G)	Young modulus (E)	Poisson ratio (σ)	Pugh ratio	Anisotropy factor (A)	Cauchy’s pressure (CP)
0	60.56	28.42	73.74	0.36	2.13	6.45	− 3.67
5	83.07	50.06	125.07	0.33	1.66	4.29	− 19.81
10	100.52	45.66	118.97	0.36	2.20	7.31	− 5.66
15	117.76	59.01	151.69	0.35	1.99	5.97	− 13.22
20	133.95	71.16	181.38	0.34	1.88	5.64	− 21.95
25	149.88	90.32	225.64	0.33	1.66	4.63	− 39.02

### Electronic features: density of states and band structure analysis

**Figure 2:**
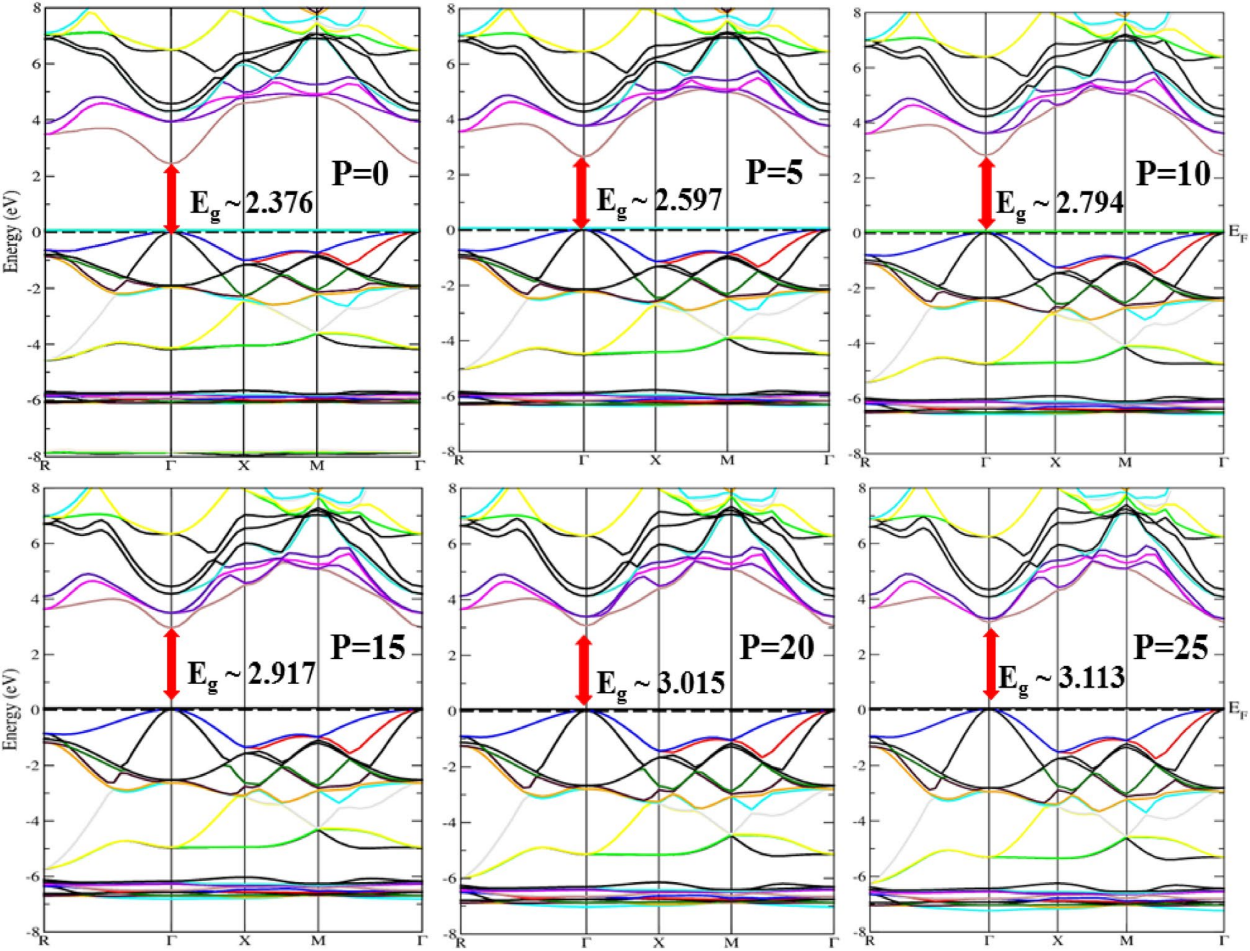
Band structures of the Cd_0.25_Zn_0.75_Se semiconductor under the influence of high pressures approximated within mBJ.

At various pressures along the path in the Brillouin zone, which has a greater number of peak symmetry points, the electronic characteristics, particularly the band structures and density of states (DOS), are analyzed. The capability of a material to examine and understand electrical behavior under pressure aids in anticipating the material’s appropriation for particular device-based applications. The influence of pressure on band structures has been illustrated using the GGA, mBJ, and EV-GGA functionals at a varying pressure range of 0–25 GPa, while the influence of pressure on lattice parameters has been initially explored using GGA and applied to band structure calculations. The band structure study shows that the Cd_0.25_Zn_0.75_Se alloy exhibits a direct bandgap nature at all applied pressure ranges. In view of the fact that GGA is commonly known for undervaluing bandgap values^36^, in order to address this underestimation of bandgap values, Engel and Vosko established a new formulation of GGA functional that yields better exchange potential and replicates electronic bandgap than GGA, and it is named EV-GGA^37^. As a consequence, we evaluated EV-GGA findings and equated them to mBJ results; nonetheless, the outcomes of these theoretical computations demonstrate that, despite the differences in bandwidths, the essential properties of a material’s band structure remain the same. The conduction band minima (CBM) and valence band maxima (VBM) are placed at similar k-space positions, indicating the nature of this alloy as a direct bandgap material, with bandgap energy ranging from 2.37 to 3.11 eV within the mBJ potential. The band structures predicted within the functionals, including GGA, mBJ, and EV-GGA, follow a similar pattern, with only a difference in the value of band splitting energy. As the pattern of the generated structures remained consistent and the overall appearance was closely matched, the band structures are solely displayed within the mBJ potential, as seen in Fig. 2.

Band structures can be deeply studied by analyzing the density of states and may also be used to determine the nature of the material, whether it is a semiconductor or a metal. Figure 1 displays the total and partial density of states for the Cd_0.25_Zn_0.75_Se semiconductor by considering the influence of the pressure effect. One can clearly observe a significant pressure influence of both the valence and conduction bands on the electron density of state (DOS); however, the pressure influence is more prominent on the electron DOS caused by the valence band electrons. The density of valence band electrons is significantly influenced by pressure, and the peak appearing at − 4 eV is displaced to a lower energy value of − 5.3 eV. Peak intensity drops as well, with the maximum peak intensity corresponding to zero pressure. In general, when pressure is applied and its impact is taken into account, the intensity of the peak’s height starts to drop as the pressure increases, while the general pattern and trend of DOS remain identical, with the only difference being a shift towards lower energies. When pressure is applied, the density of states generated is dominated by electrons in the conduction band and has no substantial influence. An inset of the conduction band near the Fermi level region’s zoom is added in Fig. 1d to demonstrate the fluctuation in bandgap value as pressure is raised, and an expansion is witnessed with an enhancement of pressure values, which confirms the increase in bandgap energy. Figure 1e,f show the PDOS of Cadmium (Cd) and Zinc (Zn) atoms at 0 and 10 GPa pressure, respectively. The PDOS of each element varies considerably, particularly in the valence band, which is characterized by Cd-s, d states, Se-s states, and Zn–s, p states, whereas the conduction band is dominated by Cd-s, p, and Zn-p states. Pressure causes the peak height in the conduction band to drop, resulting in a shift towards low energy values.

**Figure 3:**
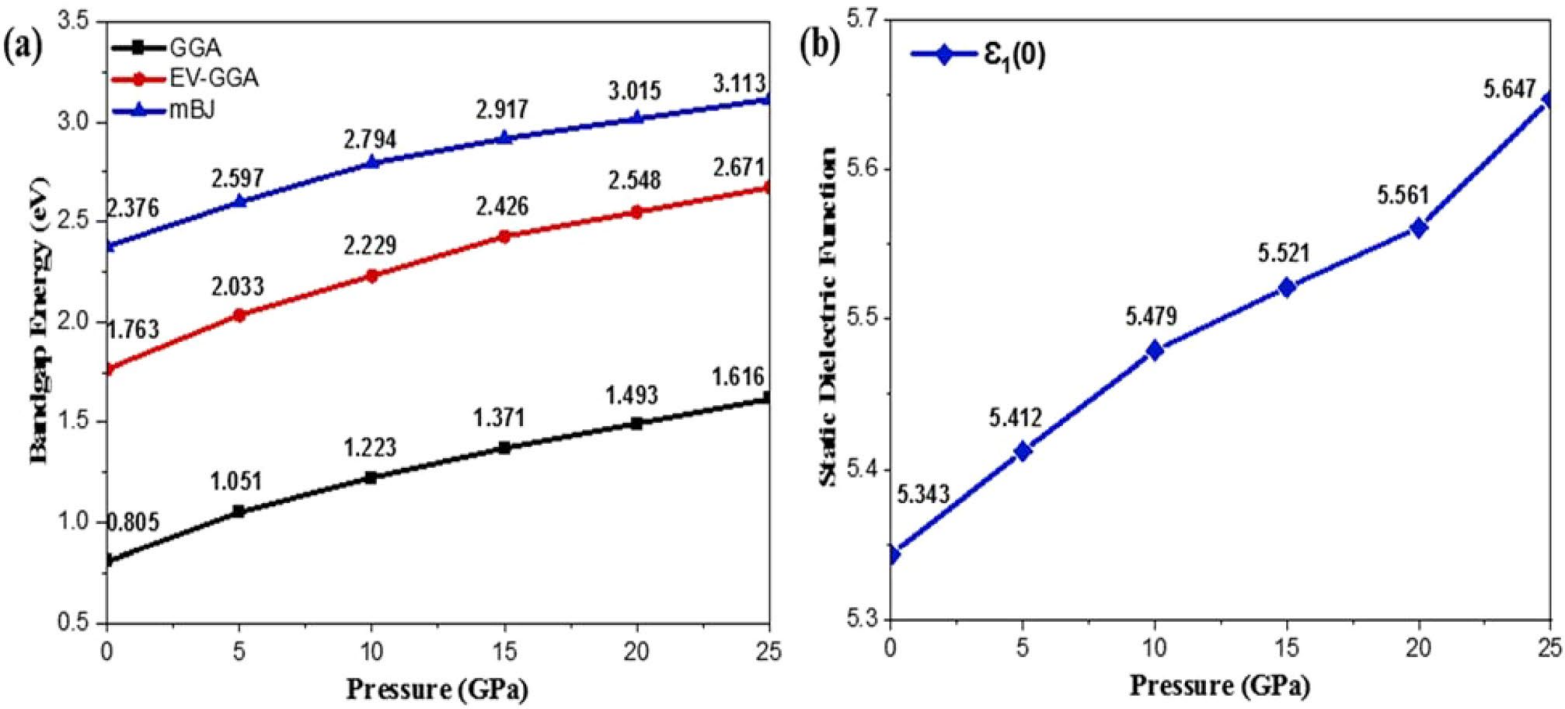
Pressure-induced variation in, (**a**) bandgap, and (**b**) static dielectric function.

Impurity bands are formed in the valence band when Cd atoms are incorporated into the supercell of ZnSe with Zn atoms, which enhances their degeneracy with increasing pressure. At high pressure, the impurity bands demonstrate the high bandgap energy by shifting towards lower energy values. Furthermore, because they are more delocalized, the valence band is observed to be more dispersed as compared to the conduction band, and pressure boosts the valence bandwidth but does not have any substantial effect on conduction bandwidth. Generally, the semiconductor material shows less ionic behavior when the bandwidth of the valence band rises, implying that the ionic character of the Cd_0.25_Zn_0.75_Se alloy decreases under pressure. The valence band maxima move in descending order by applying pressure, while the minima of the conduction band move upward, implying that the bandgap rises with pressure. These findings for band structures at zero pressure are consistent with the experimentally reported value of 2.27 eV^20^ and theoretical values of 0.76 eV and 0.83 eV^21^ approximated within GGA and LDA, providing us motivation to adjust the bandgap using better functionals to overcome GGA underestimation and to depict the feasibility of this alloy material for possible optoelectronic and photovoltaic applications. The reported bandgap of this ternary alloy shows a potential to fabricate devices that can be used in space photovoltaics under the varying temperature and pressures at specific altitude heights. Figure 3a represents a comparison of bandgap energy values computed within different functionals (GGA, EV-GGA, and mBJ).

### Optical properties

In the fields of optoelectronics and photonics, radiation-matter interactions are of great interest. When light-matter interaction occurs, the phenomena like reflection, refraction, absorption, and transmission are observed, and the material behavior is essentially governed by the illumination light frequency. The incoming photon causes an electron's transition probabilities between occupied and unoccupied states, emitting the light wavelength owing to these excitations, which may be defined using joint densities. To analyze the system's linear response, a complex dielectric function^38^ can be employed and may be written as:

10 $$\varepsilon \left(\omega \right)={\varepsilon }_{1}\left(\omega \right)+i{\varepsilon }_{2}\left(\omega \right)$$

where ${\varepsilon }_{1}\left(\omega \right)$ represents the real, and ${\varepsilon }_{2}\left(\omega \right)$ depicts the imaginary component of the complex dielectric function. This complex function provides important statistics about the polarization of the under-study material and is represented with the help of the static dielectric constant, ${\varepsilon }_{1}\left(0\right)$, which signifies the dielectric function at zero frequency. The real and imaginary components of a complex dielectric function may be approximated by following the Kramers–Kronig transformations^39^.

11 $${\varepsilon }_{1}\left(\omega \right)=1+\frac{2}{\pi }{\int }_{0}^{\infty }\frac{{\omega }^{\prime }{\varepsilon }_{2}\left(\omega \right)}{{\omega }^{\prime 2}-{\omega }^{2}}d{\omega }^{\prime }$$

12 $${\varepsilon }_{2}\left(\omega \right)=-\frac{2\omega }{\pi }P{\int }_{0}^{\infty }\frac{(\varepsilon 1({\omega }^{\prime })-1)d{\omega }^{\prime }}{{\omega }^{\prime 2}-{\omega }^{2}}$$

The joint density of states, as well as the momentum matrix are denoted by *ω*′, and P, respectively.

Dielectric function depends on incoming photon energy and can also be employed to calculate optical constants like extinction coefficient and refractive index. The refractive index is a measurement of how much light slows down as it enters a material, and it is dependent on the frequency of the incoming light^38^. The refractive index and extinction coefficient may be computed as:

13 $$k\left( \omega \right) = \frac{1}{\sqrt 2 }\left[ {\sqrt {\left\{ {\mathop {\varepsilon_{1} }\nolimits^{2} \left( \omega \right) + \mathop {\varepsilon_{2} }\nolimits^{2} \left( \omega \right)} \right\}} - \varepsilon_{1} \left( \omega \right)} \right]^{1/2}$$

14 $$n\left( \omega \right) = \frac{1}{\sqrt 2 }\left[ {\sqrt {\left\{ {\mathop {\varepsilon_{1} }\nolimits^{2} \left( \omega \right) + \mathop {\varepsilon_{2} }\nolimits^{2} \left( \omega \right)} \right\}} + \varepsilon_{1} \left( \omega \right)} \right]^{1/2}$$

The extinction coefficient may be used to govern optical medium absorption^38^, which can be utilized to analyze the light decay per unit distance in the material medium, which can mathematically be expressed as:

15 $$\alpha \left( \omega \right) = \frac{4\pi k(\omega )}{{\mathop \lambda \nolimits_{0} }} = \frac{\omega }{nc}\varepsilon_{2} \left( \omega \right)$$

The behavior of the material surface can also be examined by measuring the reflection spectrum^38^, mathematically described as:

16 $$R\left(\omega \right)=\frac{{\left(n\left(\omega \right)-1\right)}^{2}+{k}^{2}\left(\omega \right)}{{\left(n\left(\omega \right)+1\right)}^{2}+{k}^{2}\left(\omega \right)}$$

Optical conductance^38^ is a non-contact quantitative measurement which can be calculated using imaginary components of the dielectric function. The real component of the optical conductance may be expressed as follows:

17 $$\mathit{Re}\sigma (\omega )=\frac{\omega .{\varepsilon }_{2}}{4\pi }$$

The energy loss function^38^ of the electrons can be calculated as follows:

18 $$L\left(\omega \right)=lm\left(-\frac{1}{\varepsilon \left(\omega \right)}\right)=\frac{{\varepsilon }_{2}}{\left({\varepsilon }_{1}^{2}\left(\omega \right)+{\varepsilon }_{2}^{2}\left(\omega \right)\right)}$$

Penn model^40^ is utilized to determine the relationship between the material’s bandgap energy (${\mathrm{\rm E}}_{\mathrm{g}}),$ and the real component of a dielectric function $\left({\upvarepsilon }_{1}\left(0\right)\right).$

19 $${\varepsilon }_{1}\left(0\right)\approx 1+{\left(\frac{\hslash {\omega }_{p}}{{\rm E}_{g}}\right)}^{2}$$

where plasma energy denoted with $\hslash {\omega }_{p}$ is governed by ${\omega }_{p}$ (plasma frequency).

**Figure 4:**
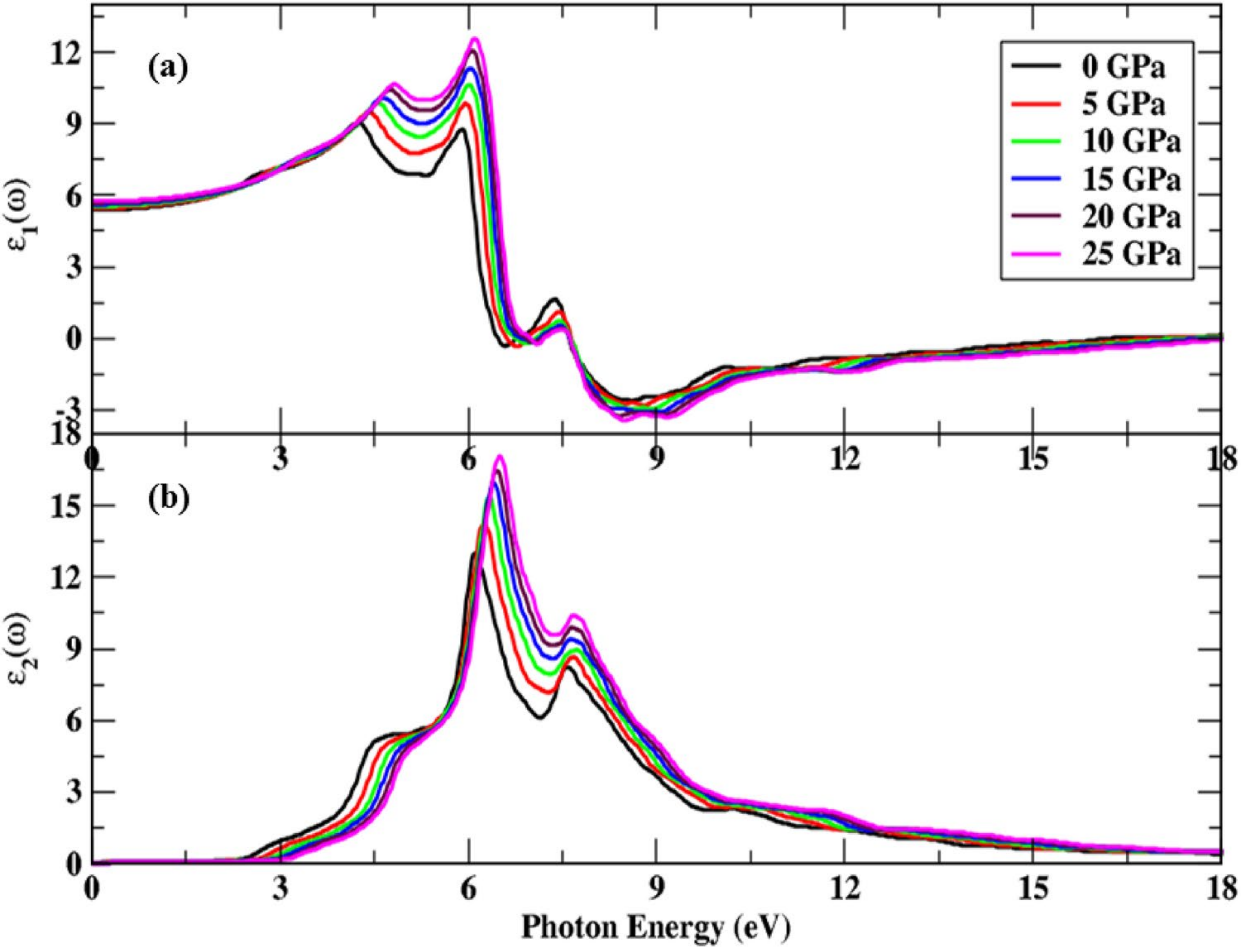
Pressure-influenced change in dielectric function, (**a**) real component, and (**b**) imaginary component.

We investigated the optical properties of the Cd_0.25_Zn_0.75_Se semiconductor for incoming light radiation of 40 eV within mBJ using a denser k-point mesh with cubic crystal symmetry for the pressure range of 0–25 GPa. The complex dielectric function is depicted in Fig. 4 and a significant increase is observed in real component of the dielectric function with a little dip and an abrupt rise to the maximum peak values can be witnessed in Fig. 4a. Moreover, the maximum transitions of 3.95–6.50 eV energy range were observed with increasing pressure and peak height, as well as a shift towards higher ${\varepsilon }_{1}\left(\omega \right)$ values was also observed. Because all incident radiation is reflected, the functional material displays metallic features below zero-unit values for illumination radiation range of 7.63–16.88 eV, proving the investigated material useful in the application of a protector for vacuum and ultra-violet radiation.

After 16.80 eV, the real component value turns positive; indicating that this semiconducting material remains unaltered in the presence of high energy incoming radiation and thus may be employed in optical lenses. However, the general drift of dielectric function remains identical regardless of the boost in peak height with pressure variation. The static index, namely, static dielectric constant, is also observed to increase with pressure impact. The energy eigenvalues corresponding to s, p, and d orbitals lie in the higher region with an increase in pressure and bandgap increases, so the lowest value for the static dielectric function at an irradiation frequency of zero corresponds to zero pressure. With the increase in pressure, the charge carrier’s mobility and the rate of hopping increase^41^. Hence, the dielectric polarization increases, causing an increase in the dielectric constant as depicted in Fig. 3b. The threshold energies of the imaginary dielectric function component are illustrated in Fig. 4b, which corresponds to the highest valence band and lowest conduction band direct interband transitions (Cd-s, d, Zn-p, and Se-s states) (Cd-s, p, and Se-p states). Above these thresholds, there is a rapid rise in imaginary component values up to 6.37 eV of incoming radiation. Moreover, three primary peaks are identified between the range of absorption threshold and 8 eV of incoming radiation, and the height of these peaks increases by increasing pressure. At zero pressure, the lowest threshold value is seen, and it rises as pressure increases. Peak height is also observed to be increased from 12.84 to 16.29 units and shifts towards higher energy values under the influence of pressure. A static spectrum is detected at 13.51 eV of incoming energy, depicting a static response of spectra to higher values of incoming radiation.

**Figure 5:**
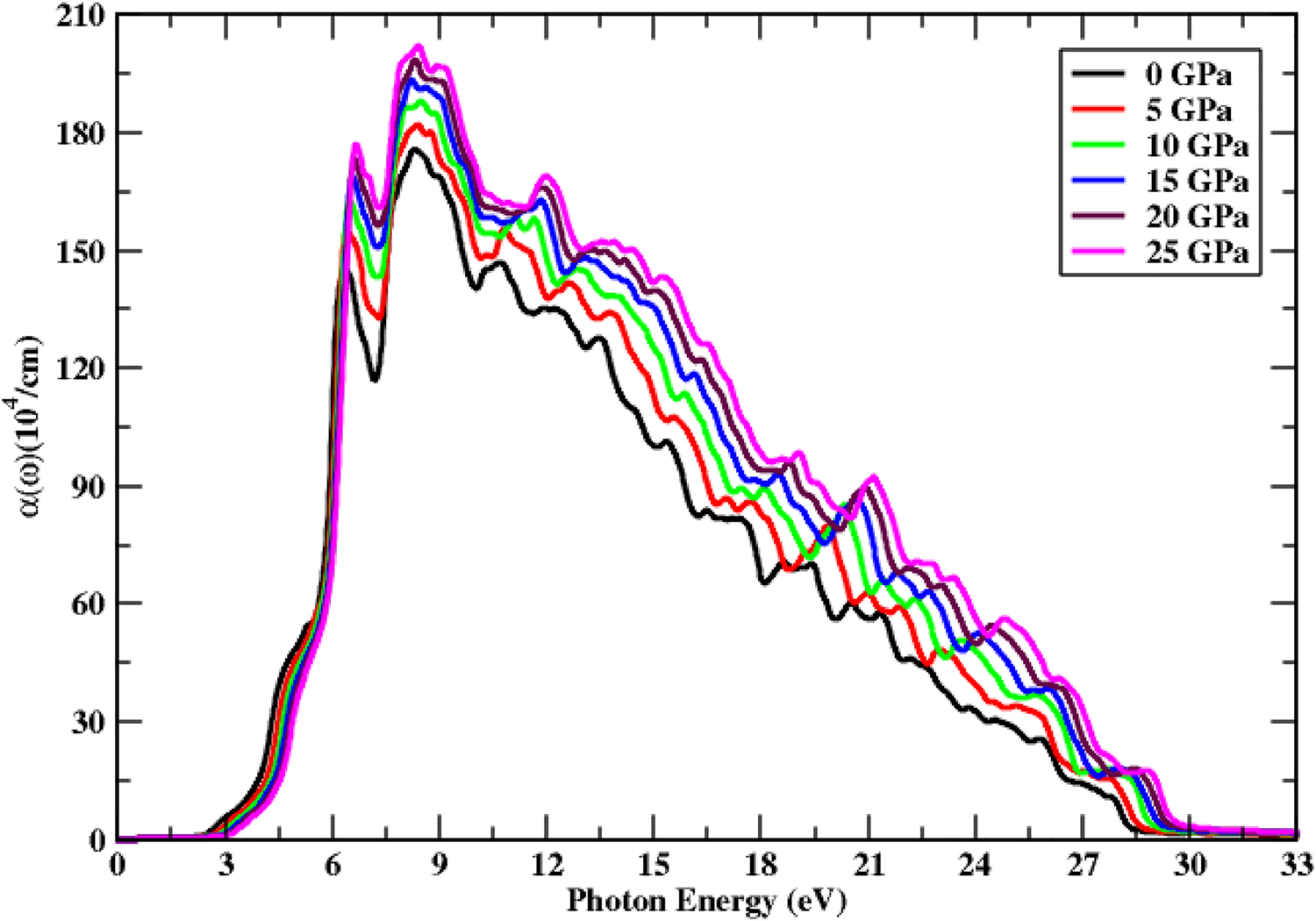
Change in absorption under the pressure’s influence.

The absorption spectrum is depicted in Fig. 5, which is strongly dependent on the dielectric function’s imaginary component $[{\varepsilon }_{2}\left(\omega \right)]$. The imaginary component of the dielectric function correlates well with the absorption coefficient. The overall pattern of the imaginary part of the complex dielectric function and the extinction coefficient, which are used to demonstrate the absorption spectrum, follow an identical pattern under the effect of pressure, so a small stepped fluctuation is observed in the absorption spectrum. Further, under the influence of the pressure, we note that the curves are similar with small shifts, which are due to the shifts of the bands only without any topology changes, and that this ternary alloy maintains its stability in the applied pressure range. The absorption rise of the functional material ranges from 174.79 to 201.28 units, with the maximum absorption peak corresponding to a pressure of 25 GPa. This material exhibits strong absorption above 8.10 eV of incoming radiation, and its absorption rises as pressure values increase. A stationary trend is recorded above 30 eV of incoming radiation; however, for lower energy photons, having less energy than bandgap energy, no absorption spectra are observed. In the energy range of 6.10–28.92 eV, higher absorption values are seen, with additional peaks resulting from valence band electronic transitions. In addition, the peak shift towards higher values is also observed in the UV region. Figure 5 shows that pressure considerably boosts optical absorption and shifts peak height towards higher values.

**Figure 6:**
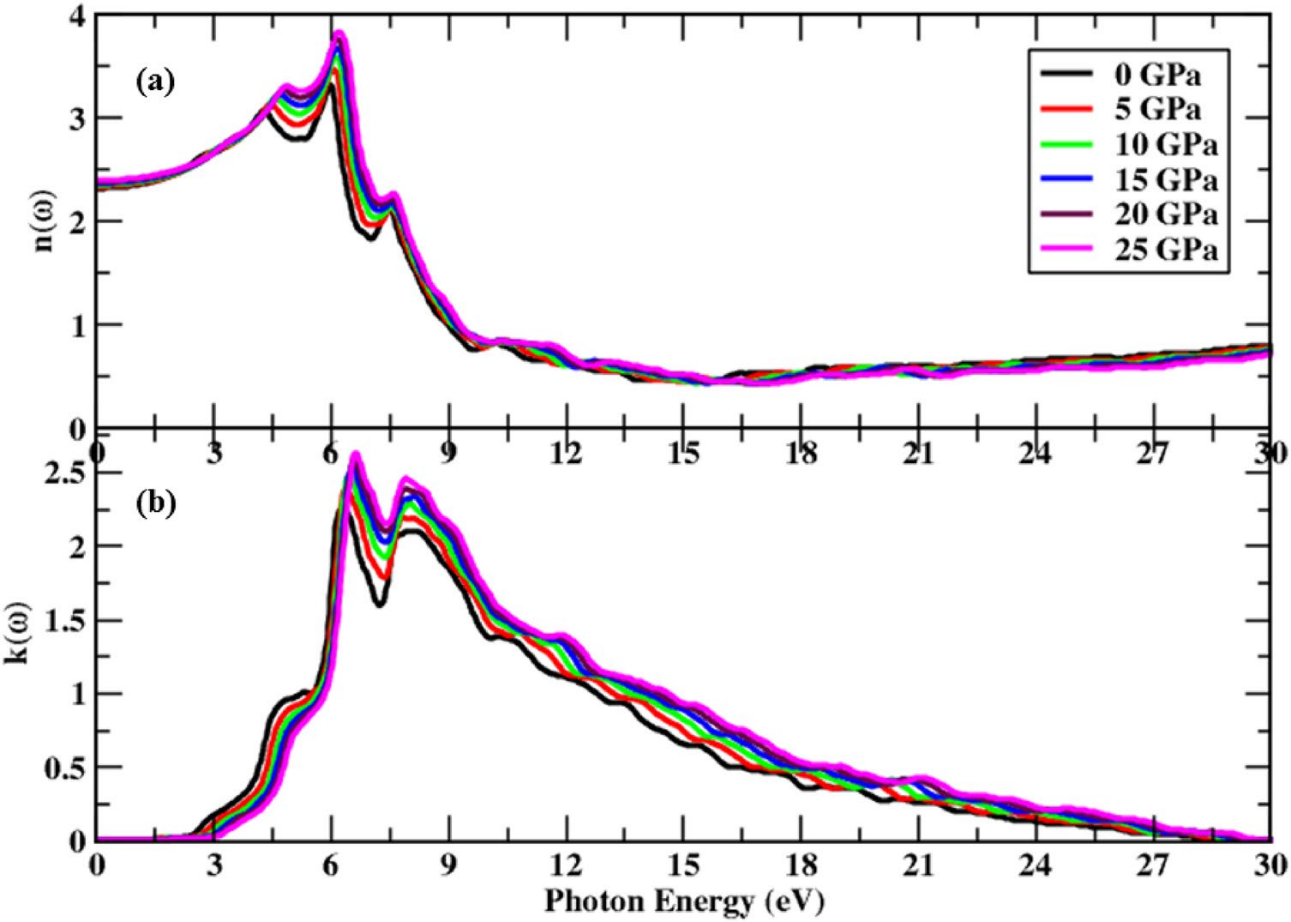
Change in complex index of refraction under pressure’s influence, (**a**) refractive index, and (**b**) extinction coefficient.

A material’s optical properties change with changing frequency; however, the under-study alloy material has cubic symmetry, hence it has the same refraction value in both transverse directions. Figure 6 shows the refractive index n(ω), which rises with increasing pressure and shows new peaks in the incoming radiation range of 4.05–7.50 eV. Figure 6a depicts the pattern of change in n(0) under the pressure impact, with values changing from 3.82 to 3.31 units, and the spectrum at 28 eV shows the material's static behavior. As the threshold energy raises the extinction coefficient, the extreme peak height likewise rises with pressure, as seen in Fig. 6b. The main peaks fluctuate from 2.59 to 2.24 units under pressure, and do not detect any spectra for extinction coefficient $k\left(\omega \right),$ beyond 29.50 eV radiation energy, since this material has no interaction with high energy photons having an energy of more than 29.50 eV, resulting in a negligible optical absorption spectrum for the incident photons. Under the pressure effect, an overall rising trend in refraction and extinction coefficient is seen, which is consistent with the findings of other optical constants as discussed above.

**Figure 7:**
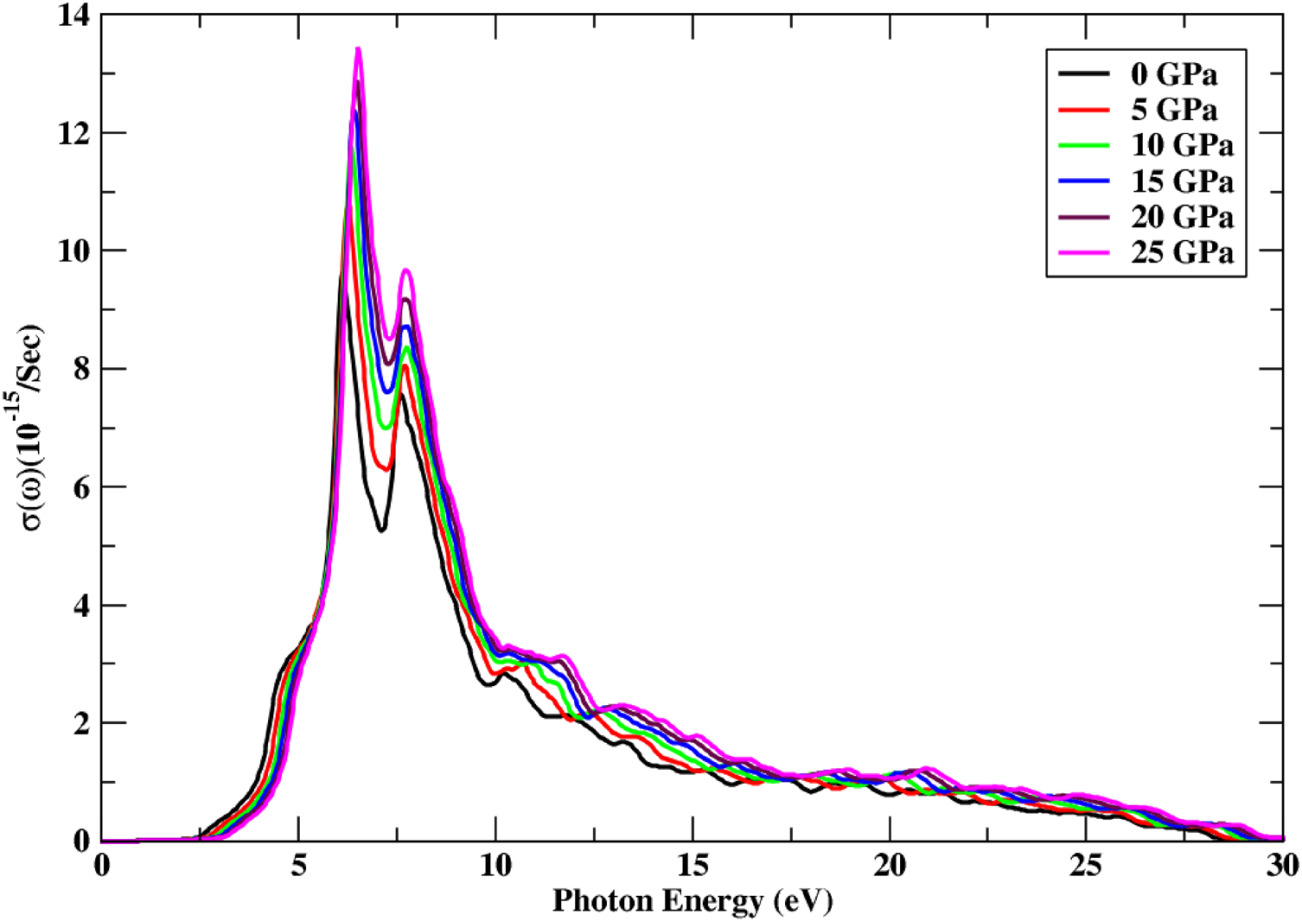
Change in optical conductance under the effect of pressure.

As long as the energy of the entering photons is smaller than the bandgap energy, optical conductance is measured to be zero. With an increase in pressure, the conductance spectrum shows a quick increase, while the peak height rises from 9.58 to 13.36 units. New peaks are found in the 5.20–10.05 eV range, with a static response at 28.22 eV of incoming radiation under the impact of applied pressure. Figure 7 depicts the change of optical conductance under the influence of applied pressure. This semiconducting material displays a high optical conductance of 13.36 units at 25 GPa, comparable to 6.59 eV of incoming radiation. It exhibits a high conductance range of 5.20–10.05 eV of incoming light, indicating that it is primarily optically active in this energy range. An increase in pressure is observed to have a direct dependence on the real part of the optical conductance.

**Figure 8:**
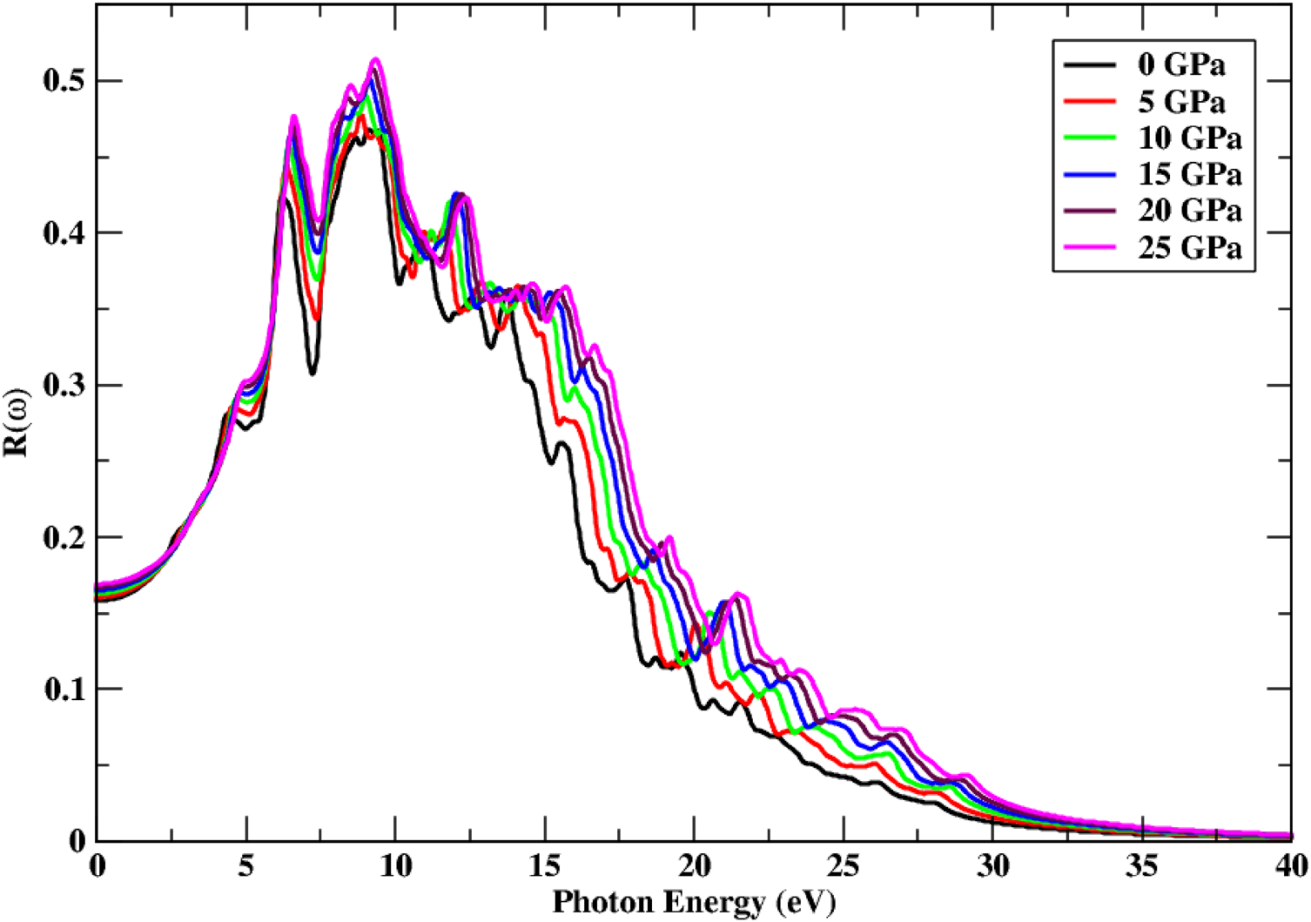
Change in reflectance under the effect of pressure.

Figure 8 illustrates an abrupt rise in reflection  with incident photon energy, as well as pressure. The shift of peaks is observed to be moving towards high range values, which is linked to the broad spectrum, while the high pressure also influences the peak height, which continues to rise. New and dispersed reflection spectra humps are detected in the incoming light range of 10.10–27.50 eV. With 0.52 values, the maximum reflectivity is measured at a high pressure of 25 GPa. The reflection value rises with pressure, and the static index of reflection increases from 0.156 to 0.167 as a result of the pressure impact. New peaks are also seen in the incoming radiation energy range of 10.11–27.50 eV.

**Figure 9:**
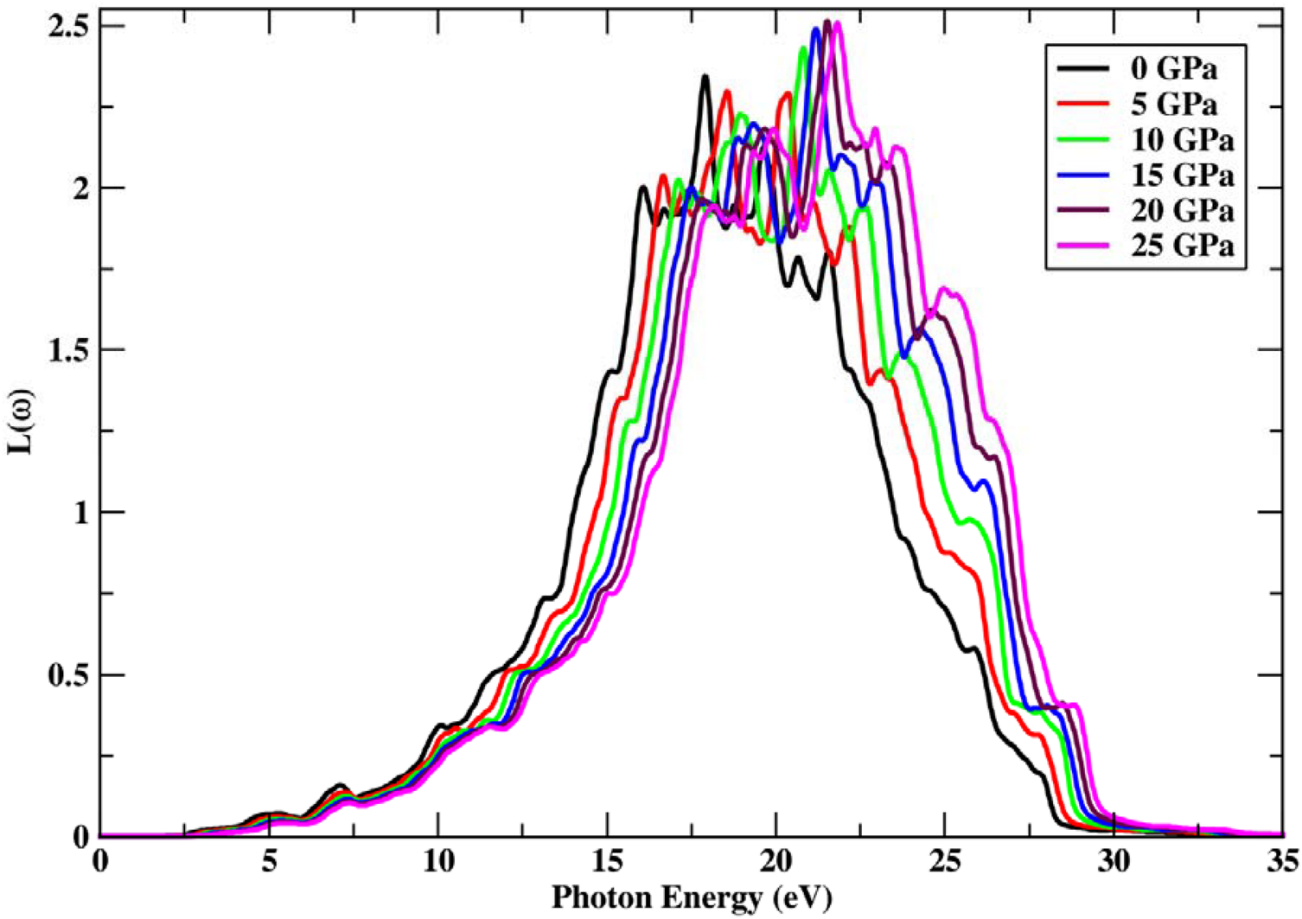
Chnage in energy loss function under the effect of pressure.

Because no electron scattering occurs at energies lower than the bandgap energy, the energy loss function is 0, and no spectra are detected. Inelastic scattering of electrons above the bandgap energy produces the L($\upomega$) spectrum, which is proportional to the incoming radiation. The highest energy loss function is observed at 25 GPa pressure with a value of 11.98 eV, as seen in Fig. 9, and it decreases as the pressure influence decreases. The electron energy loss function is reported to be very small below 4.51 eV and above 29.86 eV of incoming photon energy, depicting a static pattern. The main peaks are detected in the 10.02–28.46 eV region of incoming radiation. The peak height is seen to move towards a greater L($\upomega$) value when pressure is applied, and the highest electron energy function corresponds to higher pressure values.

## Conclusions

The present study elicited optoelectronic properties of a ternary Cd_0.25_Zn_0.75_Se semiconductor under the pressure influence using the FP-LAPW method within DFT. At all pressure ranges, this functional alloy exhibits a cubic symmetry and is mechanically stable. The semiconductor properties of the investigated material were confirmed by the band structures and density of states of the material, which show that the bandgap increases with pressure component. The width of the valence band expands, improving the covalent character and resulting in a decline in ionicity with a rise in pressure. Isotropic optical characteristics are also investigated, and the positions of critical points were detected to move towards higher energy under the influence of pressure. The peak trend is seen to be unchanged, except that the peak heights of the dielectric function, both real and imaginary parts, have increased. Optical conductance and absorption are also observed to increase under pressure, implying that this alloy can be used in the manufacturing of electronic and optoelectronic devices that function in the visible to violet light spectrum at distinct pressures and altitudes.

## Data Availability

All data generated or analyzed during this study is included in this published article.
